# Simple fluorescence optosensing probe for spermine based on ciprofloxacin-Tb^3+^ complexation

**DOI:** 10.1371/journal.pone.0251306

**Published:** 2021-05-10

**Authors:** Nguyen Ngoc Nghia, Bui The Huy, Pham Thanh Phong, Jin Sol Han, Dae Hyun Kwon, Yong-Ill Lee

**Affiliations:** 1 Department of Materials Convergence and System Engineering, Changwon National University, Changwon, Republic of Korea; 2 Ceramics and Biomaterials Research Group, Advanced Institute of Materials Science, Ton Duc Thang University, Ho Chi Minh City, Viet Nam; 3 Faculty of Applied Sciences, Ton Duc Thang University, Ho Chi Minh City, Viet Nam; Nazarbayev University, KAZAKHSTAN

## Abstract

We developed a facile detection method of spermine based on the fluorescence (FL) quenching of the ciprofloxacin-Tb^3+^ complex, which shows astrong green emission. Ciprofloxacin (CP) makes efficient bondings to Tb^3+^ ion as a linker molecule through carboxylic and ketone groups to form a kind of lanthanide coordination polymer. The addition of spermine that competes with Tb^3+^ ions for the interaction with CP due to its positive charge brings about weakened coordination linkage of CP and Tb^3+^. The probe exhibited high sensitivity, selectivity, and good linearity in the range of 2–180 μM with a low limit of detection of 0.17 μM. Moreover, we applied this method on the paper strip test (PST), along with the integration of a smartphone and Arduino-based device. The practical reliability of the developed probe was evaluated on human serum samples with acceptable analytical results.

## Introduction

Rare-earth (RE) elements have high coordination numbers, diverse modes of coordination, luminescence with a long lifetime, and narrow emission spectra. Therefore, they have been widely used for fabricating coordinated complexes with a distinct luminescent property for applications in optical sensing systems. Especially, their fluorescence intensity could be amplified through the formation of coordinated complexes, in which organic ligands play a role as an antenna of light absorption. As a result, the choice of suitable RE ions and organic ligand/linker can lead to creating desired coordination complexes with excellent luminescent properties [1–3]. Among these RE ions, terbium (Tb^3+^) ion is often used to build luminescent coordination complexes because it has a large radius, high affinity to oxygen-nitrogen hybrid compounds, and flexible coordination geometry. Intramolecular energy transfer from the excited triplet states of the ligand to Tb^3+^ ions in chelate results in an intensive fluorescence with a large stoke shift. Some coordination complexes of Tb^3+^ have been proposed as fluorescent probes for sensing small organic molecules, aromatic compounds, ions, and vapors [4–13]. Altunbas’ group [4] suggested Ochratoxin A (OTA) sensor based on the coordination of OTA to Tb^3+^ ions, which were attached to the EDTA modified SiO_2_ nanoparticles, resulted in the enhancement of the fluorescence intensity of Tb^3+^. Anwar’s group [5] developed Al^3+^ sensor based on the fluorescence enhancement of Tb^3+^-3,4-dimethyl-thieno[2,3 b] thiophene-2,5-dicarboxylic acid complex. Using the antenna effect of RE-metal-organic frameworks (MOF), Zhang et al. [7] synthesized Tb^3+^-based on MOF, where 4-(pyridine-3-yloxy)-phthalic acid and oxalic acid as organic linkers with Tb^3+^ ions for detecting Al^3+^ and CO_3_^2-^. A porous network polymer was coordinated with Tb^3+^ ions to create a ratiometric fluorescent sensor for detecting dipicolinic acid, in which dipicolinic acid played as an “antenna” and transferred its absorbed energy to Tb^3+^ ions to result in enhancement of Tb^3+^ fluorescence intensity [8]. Using the aggregation-induced fluorescence of Tb^3+^- luminol as an “electron donor” to uric acid, Qi’s group suggested a uric acid sensor based on the photoinduced electron transfer effect to result in a quenching of fluorescence intensity of the complex [12].

Spermine (SP), a polyamine presents in fluids and eukaryotic cells of many living organisms. Decarboxylation of amino acids by enzyme forms SP and other biogenic polyamines as well. SP plays a significant role in cell growth, proliferation, and signal transduction. The increased level of SP concentration in urine relates to the presence of malignant tumors to indicate cancer remission or relapse [14, 15]. Moreover, spermine is also considered as one of the key trigger factors in food toxicity and can be used as a food quality indicator [16]. However, its low volatility, low molecular weight, and the absence of chromophores significantly impede the sensitivity and selectivity for SP detection [17–20]. Conventional methods for the quantitative determination of spermine, such as chromatographic techniques [21–23], immunoassays [24, 25], electrophoresis [26–28], and electrochemical [29, 30] have been developed, but the time consuming, skilled personnel, and high cost of related equipment significantly affect the popularity of these methods. In contrast, fluorescence methods have become a popular method due to the versatility, sensitivity, low detection, and visual capacities [31–33]. For example, Fletcher and Bruck [34] reported an SP sensor based on “turn-on” fluorescence of dicarboxylated ethynylarene by mixing Pb(II) cations with SP. However, the preparation procedure of dicarboxylated ethynylarene was too complicated to adapt for practical applications. Satrijo and Swager [35] found that the blue emission of polyanionic poly(p-phenylene ethynylene) changed to green in the presence of SP as a result of enhanced exciton migration. Jiang’s group proposed a supramolecular compound as a fluorescent sensor, which was synthesized from luminogen and cucurbit [7] uril, for detecting SP with aggregation-induced emission [36]. Fukushima and Aikawa [37] suggested a spermine probe based on the color change of mixture pyrocatechol violet and carboxyphenyl boronic acid. Naik’s group used the aggregation-induced-emission effect of tetraphenylethylene derivative, which was conjugated with cucurbit [6] uril hydroxyl, and hydroxyapatite surface nanoparticles, resulting in an enhancement of FL intensity in the detection of spermine [38]. Although some techniques exhibited a low limit of detection, they were performed through organic synthetic processes that require plenty of time, high cost of precursors, and skilled labor.

Ciprofloxacin (CP) is a type of antibiotic commonly used containing a configuration of α-carbonyl carboxylic acid, which is suitable for coordinating with a RE ion. Among these coordination complexes, Tb^3+^ ions exhibit high effective reactivity with CP [39–42]. To the best of our knowledge, the fluorescent probe for sensing SP using the coordination of Tb^3+^ and ciprofloxacin is not reported yet. As an analytical platform, paper-based analytical devices are commonly used because PSTs have many beneficial properties such as simple, lightweight, safe incineration disposal, handy, and cost-effective analytical devices [43–46]. Arduino is an open-source electronic platform based on easy-to-use software and hardware for building digital devices. Arduino board can integrate into a variety of microprocessors and controllers and be programmed using “Arduino language” to fabricate various projects. Recently, numerous researchers have used Arduino as color reader, voltmeter, and barometric controller in biotechnology applications [46–49].

**Scheme 1:**
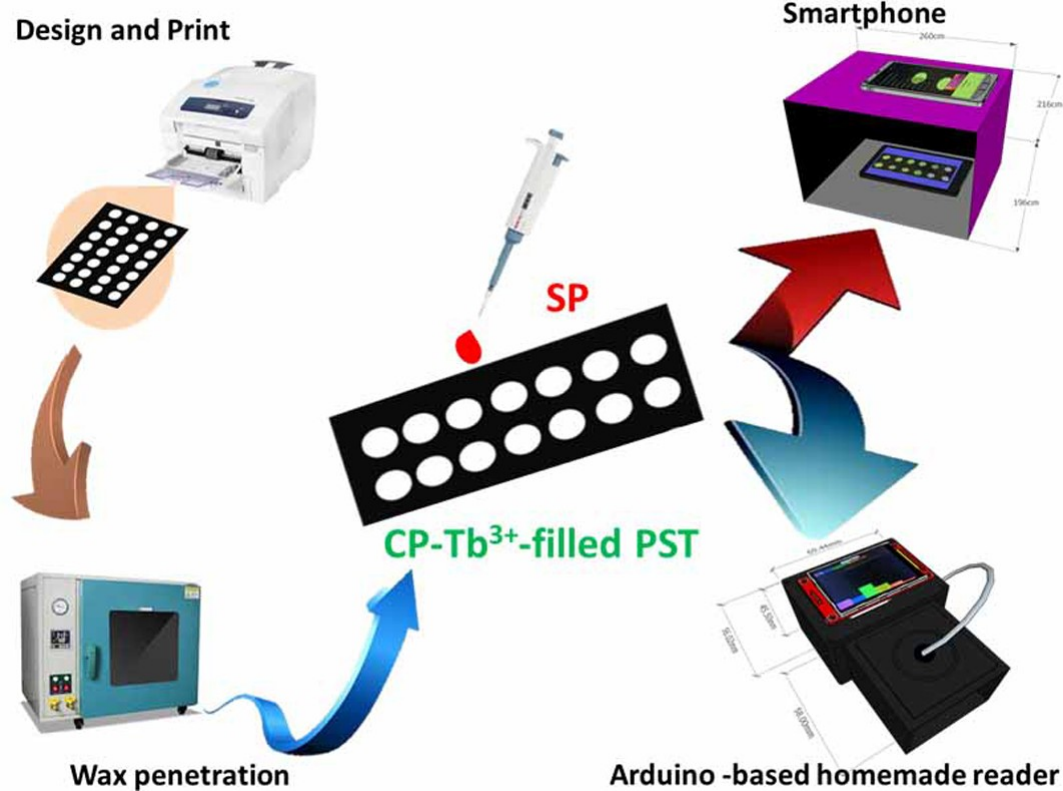
Illustration of the experimental procedure for detecting spermine. Smartphone and Arduino device were used as fluorescence readers.

In this work, a novel paper strip-based system is developed with simple and high efficiency for sensing SP based on fluorescence quenching of CP-Tb^3+^ complex by SP. Detection areas on paper strip test (PST) were isolated by wax ink and filled with CP-Tb^3+^ solution. The amount of SP was determined by monitoring the change of color strength, initiating from the fluorescence quenching of the CP-Tb^3+^ complex. Moreover, we prepared an Arduino-based device as a reader based on AS7262 6 channels VIS sensor (S1 Fig in S1 File) with a specific circuit and design. The details of the homemade reader are presented in S1 File. Data were collected either on the homemade reader or smartphone. The procedure for preparing the SP sensor is illustrated in Scheme 1. In brief, PST was printed by using a wax printer. The detection areas on the PST were filled with CP-Tb^3+^ complex as a fluorescence indicator after the wax penetration process was completed in an oven. After adding spermine samples, the FL intensity of CP-Tb^3+^ complex was recorded by using either a smartphone or Arduino-based homemade reader.

## Materials and methods

### Reagents

Ciprofloxacin, Terbium(III) oxide, hydrochloric acid (37%), poly(ethylene oxide) (PEO) (average Mv 600,000), human serum (from human male AB plasma), and spermine were purchased from Sigma-Aldrich (Korea). Deionized water purified with Millipore was used throughout the whole experiment. Terbium(III) oxide (0.1 mmol) was dissolved in dilute hydrochloric acid at 90°C for 1h. The mixture was evaporated using a rotary evaporator and then dried at 50°C in a vacuum oven. Finally, the final product was dissolved in 10 ml of H_2_O to make Tb^3+^ precursor solution.

### Design of paper strip test

PSTs consist of circles with a diameter of 0.71 cm and line weight of 4^1/2^ pt_,_ designed by Illustrator software and printed on the Whatman filter paper grade #1 by a Xerox ColorQube 8570DN. The wax was penetrated completely into the paper after heating at 60°C for 7 minutes. The precursor solution of 500 μl of Tb^3+^ solution (10 mM), 5 ml of CP (1mM), and 150 mg of PEO were mixed under vigorous stirring for 2h. 10 μl of the mixture was dropped on PSTs, and then dried for 15 min to get PST-SP.

### Characterizations

**Fig 1:**
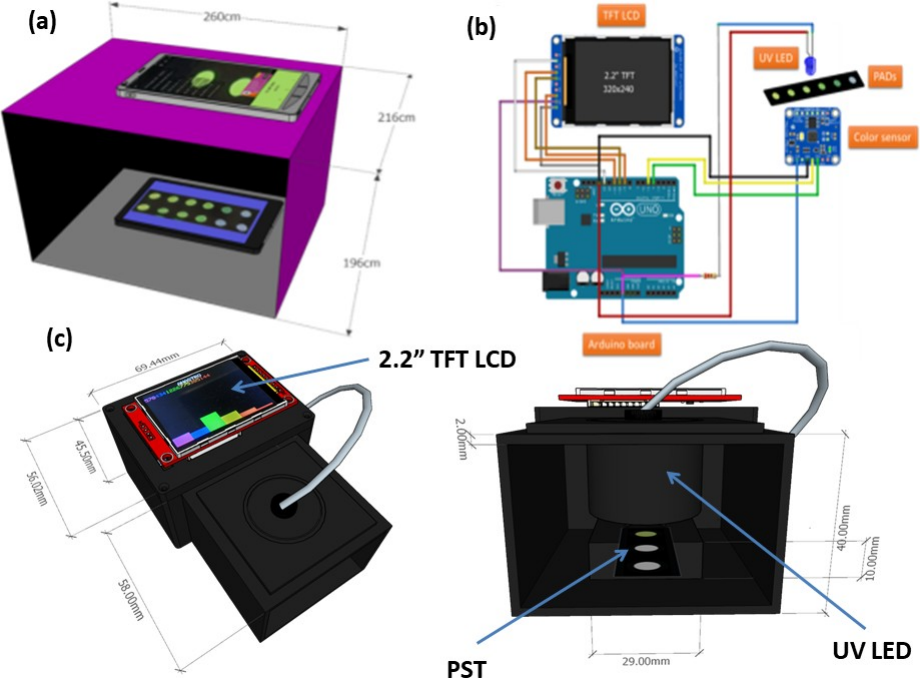
(a) Design sketch for SP detection using a smartphone; (b) Electrical circuit of Arduino-based device; and (c) Specific design of home-made reader: AS7262 6-channel VIS sensor, UV-LED 365 nm as an excitation source, 2.2” thin-film transistor liquid crystal display (TFT LCD), and controller board ATmega-328P microcontroller Arduino Uno R3.

The absorption spectra were recorded by using a UV/Vis spectrophotometer (Agilent 8543, USA). Fluorescence spectra were obtained with an FP-6500 spectrofluorometer (JASCO, Tokyo, Japan) under an excitation of 365 nm wavelength. 20 μl of SP solutions with various concentrations have been dropped into circles on the PST. After 15 min of reaction, the PST was exposed to a UV 365 nm lamp (Spectroline/UV Hand Lamp 4W/ENF-240C/FE) in a dark box (26 x 21.6 x 19.6 cm^3^), and the color images of the PST were captured by a smartphone (Galaxy Note 9 with Superspeed Dual Pixel 12 MP AF sensor, a dual aperture of F1.5 mode/F2.4 mode). The color images were analyzed to hue values using the color Picker app. Instead of using a smartphone, the SP dropped PST was inserted into a homemade reader, which consists of a UV-LED (365 nm wavelength) and AS7262-6 channels visible light sensor with 6 channels for recording the color signal with digital form. Fig 1A displays a sketch for SP detection using a smartphone. Fig 1B and 1c show an electric circuit of an Arduino-based device and the design of the Arduino-based homemade reader, respectively. The details of the homemade reader can be found in S1 File.

### Compliance with ethical standards

No violation of human or animal rights occurred during this investigation. All experimental procedures were performed in compliance with the relevant laws and institutional guidelines, and approved by the ethics committee at Changwon National University, Korea.

## Results and discussion

### Principle and sensing mechanism of spermine detection

**Fig 2:**
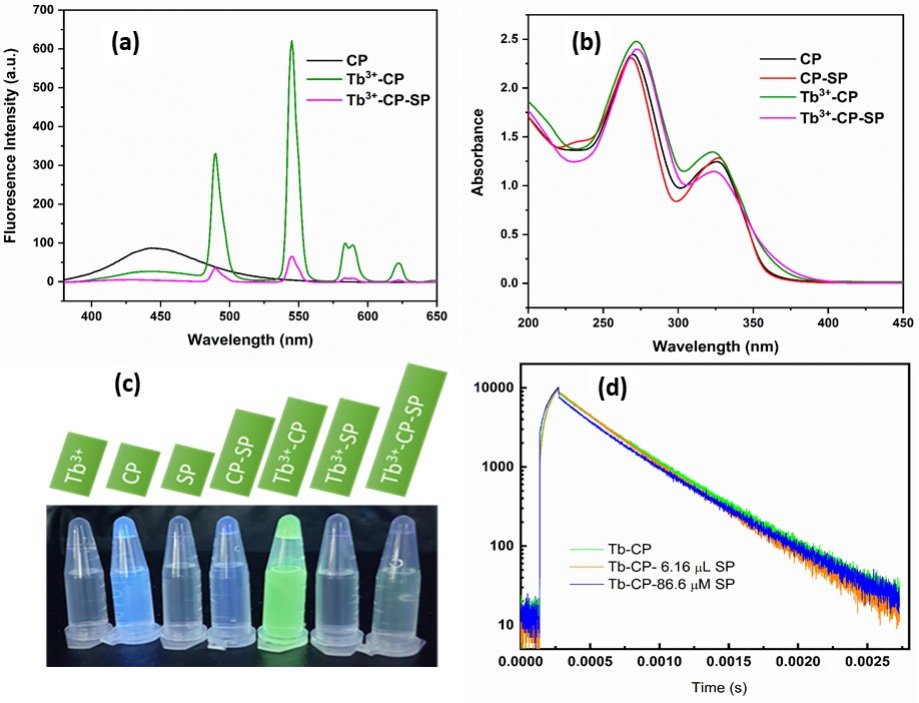
(a) Fluorescence and (b) UV spectra of CP, CP-SP, Tb^3+^-CP, Tb^3+^-CP-SP and Tb^3+^-SP samples with CP (0.13 mM), SP (74 μM), Tb^3+^ (0.02 mg/mL); (c) Color changes of the samples under UV lamp (λ_max_ = 365 nm); and (d) Life-time curves of Tb-CP, Tb-CP-6.16 μM SP, Tb-CP-86.6 μM SP samples.

The emission spectrum of Tb^3+^ ions consists of two strong peaks located at 490 nm and 545 nm, corresponding to ^3^D_4_-^7^D_6_ and ^3^D_4_-^7^D_5_ transitions of Tb^3+^ ions, respectively [50]. In our optimized conditions, the fluorescence intensity of Tb^3+^ ions was relatively weak, but it was significantly enhanced when coordinated with CP, as shown in Fig 2A, by forming six-membered chelation through the coordination between Tb^3+^ ions and 3-carboxyl and 4-keto to lead the intramolecular energy transfer from the ligand to the central Tb^3+^ ions [51]. The increase of FL lifetime of ^3^D_4_-^7^D_5_ transitions (545 nm) of Tb^3+^ ions (~ 0.04 ms) and Tb-CP complex (~ 0.37 ms) was also observed, which can be explained by reducing the fluorescence quenching effect, and by increasing the absorbance energy transfer emission through successful coordination between Tb^3+^ and CP [52]. Studies on absorption spectra showed that the absorption peaks of the CP-Tb^3+^ complex were shifted, and the absorbance was enhanced compared with that of the CP compound by adding Tb^3+^ (Fig 2B). This indicates that CP can form a binary complex with Tb^3+^. Besides that, we can see clearly that the characteristic fluorescence peak of Tb^3+^ at 545 nm in the CP-Tb^3+^-SP complex was quenched remarkably compared with CP-Tb^3+^ after the addition of SP (Fig 2A), implying that SP can form a ternary interaction with CP-Tb^3+^ system. It is known that SP has four positive charges, so it supposes that there is a binding competition between SP and Tb^3+^ to CP [53]. The color change and depletion of the compounds are displayed in Fig 2C.

The mechanism of fluorescence quenching is often explained by static quenching or dynamic quenching [54, 55]. In the static quenching mechanism, FL intensity is decreased without changing the lifetime of excited state. In opposite, dynamic quenching results in a decrease of lifetime with increasing quencher concentration. As seen in the absorption spectra of CP and CP-SP complex, they had no overlap with fluorescence emission/excitation peaks of Tb^3+^ ions. Therefore, the inner filter effect could be excluded. The absorption spectra of CP and CP-SP compounds indicated that new UV-vis absorption bands were not observable with the addition of SP into the CP (Fig 2B). As a result, the static quenching mechanism can be excluded because of the absence of a new ground state complex with a unique absorption. The lifetime values of the excited ^3^D_4_-^7^D_5_ transitions of Tb-CP samples were determined by the FL decay curves (Fig 2D). The calculated average lifetime values decreased from 0.37 ms to 0.34 ms and 0.05 ms in the presence of 6.18 μM and 86.63 μM of SP, respectively, to indicate the presence of the dynamic quenching. In addition, the zeta potential values of CP (0.13 mM), CP-Tb^3+^ and CP-Tb^3+^-SP (at Tb^3+^ (54 μM) and SP (74 μM)) samples were measured to be -7.57 mV, 3.62 mV and 40.8 mV. The initial negative zeta potential of CP transformed into positive after adding Tb^3+^ ions, indicating that the positive charges on the surface of CP were neutralized by Tb^3+^ ions due to the formation of CP-Tb^3+^ complex. Furthermore, it was increased significantly from 3.62 mV to 40.8 mV in the presence of SP. Based on these results, we believe that SP interacts with CP-Tb^3+^ via electrostatic interaction and then induces electron transfer, resulting in dynamic quenching in the fluorescence of Tb^3+^-CP.

FT-IR studies on the samples were conducted, as displayed in S2 Fig in S1 File. FT-IR spectra of CP show the peaks located at 1650 to 1600 cm^-1^ assigned to quinolones while the bands at the 1450 to 1400 cm^-1^ related to ν_C-O_. The peaks in the range of 1300–1250 cm^-1^ are suggested bending vibration of the O-H group, which indicated the presence of carboxylic acid [56, 57]. These bands are blue-shifted in the presence of Tb^3+^ to indicate Tb^3+^ ions could coordinate with the carbonyl group and protonated carboxylate. After the addition of SP, no change of vibration appears.

### Optimization of the experimental conditions

**Fig 3:**
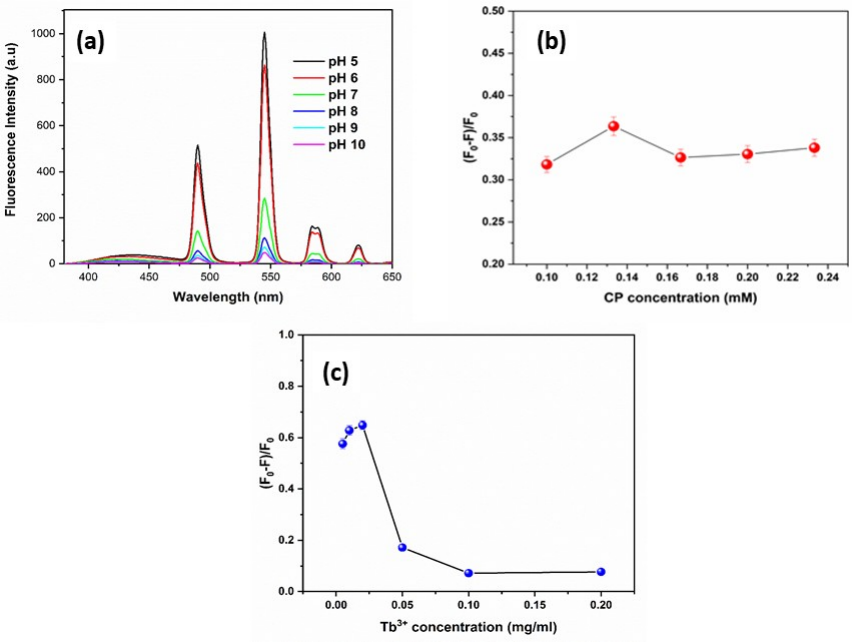
(a) Effect of pH on the fluorescence of CP-Tb^3+^ with CP (0.13 mM), Tb^3+^ (0.02 mg/mL); (b) Effect of CP concentration to the quenching efficiency of CP-Tb^3+^ in the presence of SP with SP (37 μM), Tb^3+^ (0.05 mg/mL); and (c) Effect of Tb^3+^ concentration to the quenching efficiency of CP-Tb^3+^ in the presence of SP (37 μM), CP (0.13 mM).

Experimental parameters such as the concentration of (CP, Tb^3+^) and pH have been investigated to get an optimum condition for SP sensing. As shown in Fig 3A, the FL intensity of CP-Tb^3+^ decreased from pH of 5 to 10, due to the formation of insoluble terbium hydroxide [58, 59]. In order to apply the proposed method to real samples, pH 7 was chosen for further experiments in consequence of physiological pH. The effect of CP concentration on the quenching efficiency of SP on the CP-Tb^3+^ system was estimated (Fig 3B). The maximum quenching efficiency was observed at the CP concentration of 0.13 mM when Tb^3+^, SP concentrations were 0.05 mg/mL, 37 μM, respectively. The concentration of Tb^3+^ was also investigated and optimized. The quenching efficiency increased to the Tb^3+^ concentration of 0.02 mg/ml before dropping significantly, as shown in Fig 3C.

### Detection of spermine

**Fig 4:**
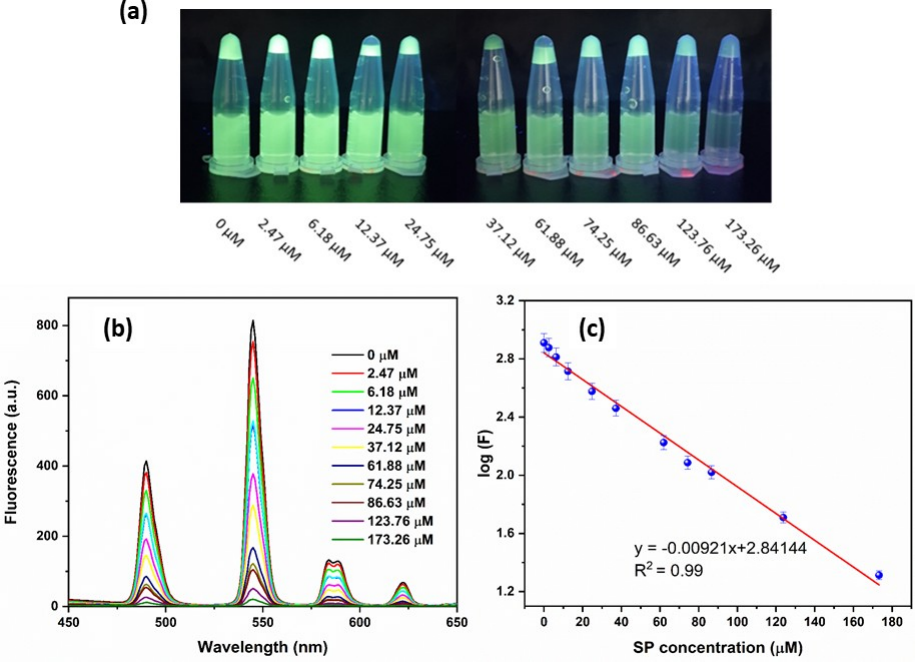
(a) Images of CP-Tb^3+^ complex with different concentrations of SP; (b) Fluorescence spectra of CP-Tb^3+^ with different concentrations of spermine (0–173 μM) in PBS buffer solution (pH 7); and (c) Linear relationship between log(F) and spermine concentration.

The pictures of the CP-Tb^3+^ complexes with different SP concentrations under UV light are displayed in Fig 4A. The fluorescence intensity of CP-Tb^3+^ decreased gradually with increasing the SP concentration, as shown in Fig 4B. The regression equation of the relationship between [SP] and the log(F) was found to be log(F) = -0.00921[SP] + 2.841 with R^2^ = 0.99, where [SP] and F are the SP concentration in μM, and fluorescence intensity, respectively (Fig 4C). The limit of detection (LOD) for SP was calculated to be 0.17 μM arcording to the 3.3 SD/S (SD, and S are standard deviation of the signal, and slope of the calibration curve, respectively) with the linear range of 2.0–180 μM.

**Fig 5:**
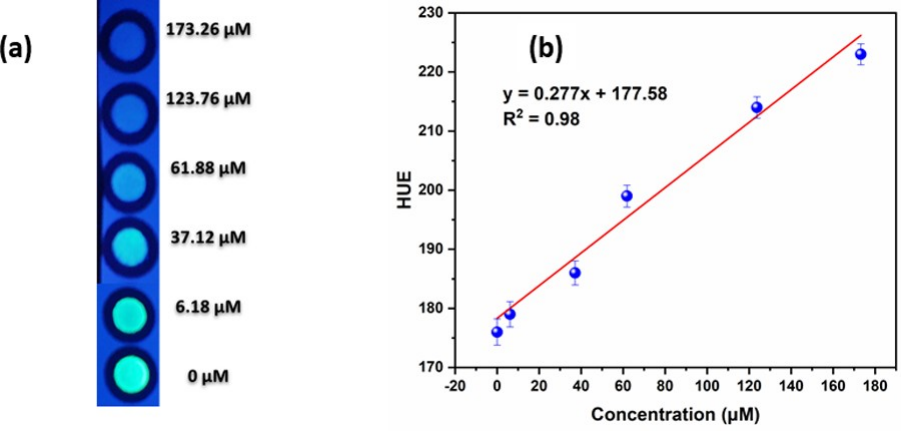
(a) Color changes of PSTs captured by smartphone (b) Relationship between hue values extracted from Color Picker App and spermine concentrations.

The values of SP concentrations obtained with a smartphone or a home-made reader were compared to evaluate the availability of recording method and signal processing technique by using a smartphone or a home-made reader for detecting SP. The SP solutions (20 μL) with different concentrations were dropped on PSTs, and then PSTs were exposed to UV light (λ_max_ = 365 nm). The color images were captured by smartphone and analyzed to hue value via Color Picker App (Fig 5A). The relationship between the hue intensity and the concentrations of SP in the range of 6.2–180 μM was fitted (Fig 5B) with the linear equation F = 0.277[SP] + 177.58 with R^2^ = 0.98, where [SP] is the SP concentration (μM). The LOD was calculated to be 1.3 μM with the smartphone.

**Fig 6:**
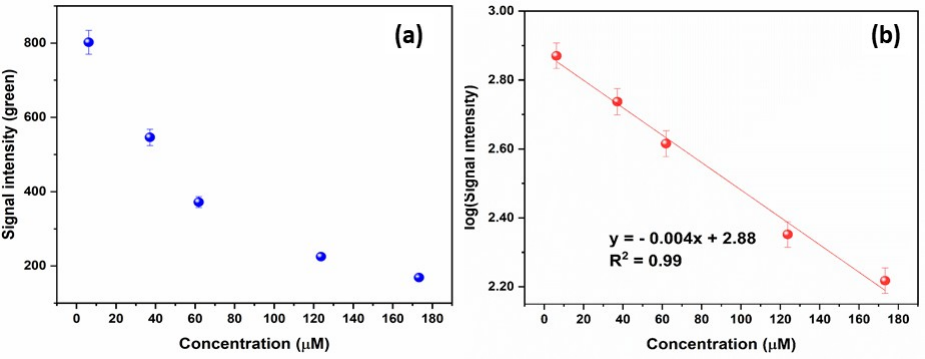
(a) Signal intensity recorded from Arduino device based on home-made reader vs SP concentration; (b) Relationship between log(S) and spermine concentration.

The Arduino-based device was used to record the fluorescence intensity on PSTs using a home-made reader. The AS7262 spectral sensor can detect 6 channels: 450 nm (Violet), 500 nm (Blue), 550 nm (Green), 570 nm (Yellow), 600 nm (Orange), and 650 nm (Red). We used the green channel for collecting data. The dependence of the recorded signal (S) on the SP concentration is expressed with the regression equation of log(S) = -0.004[SP] + 2.88, where [SP] is the SP concentration (μM), as shown in Fig 6. The LOD was calculated to be 3.3 μM in the SP range from 6.2 to 173 μM using an Arduino-based device.

Compared with other methods, our developed approach exhibits comparable or better sensitivity, selectivity, and potential to apply in biological samples (Table 1).

**Table 1:** Comparison of various analytical methods for spermine detection.

Probe	Method	Medium	pH	LOD (μM)	Reference
Dicarboxylated ethynylarene	Fluorescence	Aqueous solution	7.6	25	[34]
Anthryl-Doped Conjugated Polyelectrolytes	Fluorescence	EtOH: H_2_O 1:1	5.5	0.69	[35]
Supramolecular system (1@CB[7])	Fluorescence	Aqueous solution		1	[36]
Pyrocatechol violet and 3-carboxyphenyl boronic acid	Colorimetric		7.0	6.24	[37]
Tetraphenyl ethylene conjugated pentiptycene	Fluorescence	Water with 1% MeOH	7.4	0.3	[60]
Pb-mediated ethynylarene	Fluorescence	Aqueous solution	7.6	25	[34]
A carboxylic acid-functionalized polyfluorene (PFCOOH-BT5)	Fluorescence	DMSO	7	2	[61]
Ciprofloxacin@Tb^3+^	Fluorescence	Aqueous solution	7	0.17	This work

### Selectivity

**Fig 7:**
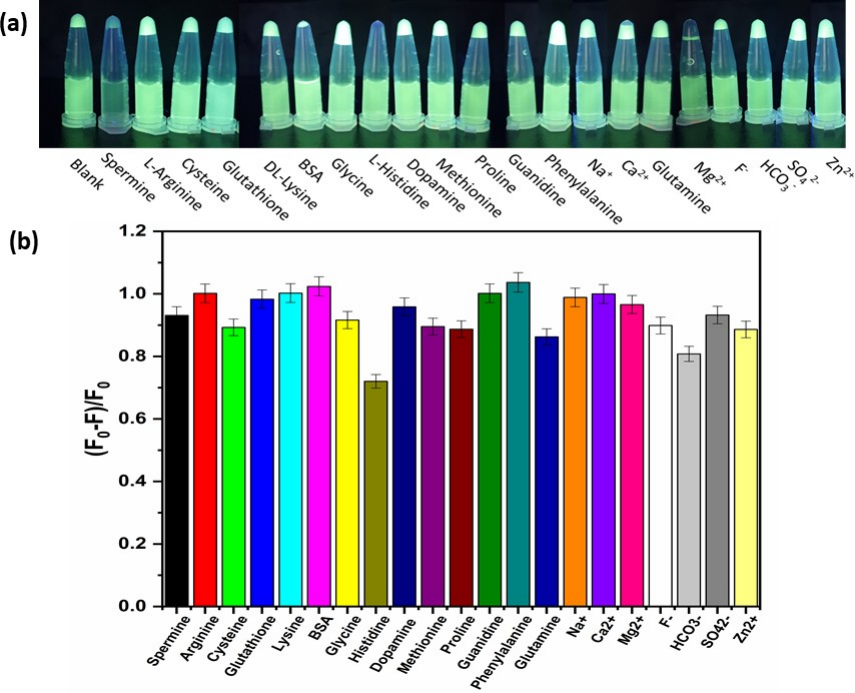
(a) Images of the samples under an explosion of UV lamp exhibited that FL of CP-Tb^3+^ complex was strongly quenched by SP (0.1 mM) as compared with available interferences (1 mM); (b) Effect of available interferences on (F_0_ –F)/F_0_ ratios of CP-Tb^3+^ in the simultaneous presence of SP (0.1 mM) and another compound (1 mM) (F_0_, F are FL intensity of CP-Tb^3+^ complex in the absence and existence of analytes, respectively).

The selectivity of the developed sensor for SP detection and interferences were examined with typical concomitant ions (Na^+^, Ca^2+^, Mg^2+^, F^-^, HCO^3-^, SO_4_^2-^, Zn^2+^) and analogous compounds including L-Arginine, Cysteine, Glutathione, DL-Lysine, BSA, L-Histidine, Dopamine, Methionine, proline, guanidine, phenylalanine, and glutamine. The fluorescence of CP-Tb^3+^ in the simultaneous presence of SP (0.1 mM) and other interferences at a concentration of 1 mM changed with a negligible value as compared with that of the CP-Tb^3+^-SP sample, as shown in Fig 7. The selectivity of the proposed method to spermine can be explained by the specific structure of spermine with four positive charges compared to other analogous compounds, which is discussed in detail in the mechanism part. These results validated the high selectivity of the proposed method.

The potential of the proposed sensor for practical applications was evaluated with a human serum sample. 20 μL of SP standard solutions with different concentrations was added into the 2 mL of the mixture of human serum samples, which was diluted by 50-fold with phosphate-buffered saline (PBS - 10 mM, pH 7), and CP-Tb^3+^ (CP-0.13 mM, Tb^3+^- 0.02 mg/mL). The fluorescence spectra of the samples were recorded after 15 min. Table 2 displays the recoveries of human serum samples containing different concentrations of SP. The results displayed that the recoveries of SP reached from 83.9% to 117.5% through three detection systems; fluorospectrometer, smartphone, and homemade reader. These results confirm that the developed probe can be applied for detecting SP in biological samples.

**Table 2:** Determination of SP in serum samples by three different detection systems.

Method	Added (μM)	Founded (μM)	Recovery (%)	RSD (%, n = 3)
	2.47	2.44 ± 0.04	98.8 ± 2.3	1.48
Fluorospectrometer	6.18	6.09 ± 0.14	98.5 ± 3.2	2.28
	12.3	12.46 ± 0.22	101.3 ± 2.5	1.73
	6.18	6.77 ± 0.13	109.5 ± 2.9	2.04
Smartphone	37.12	34.46 ± 0.41	92.8 ± 1.6	1.19
	61.88	58.84 ± 1.41	95.1 ± 3.2	2.42
	6.18	5.79 ± 0.13	93.7 ± 2.9	2.27
Home-made reader	37.12	31.16 ± 0.74	83.9 ± 2.8	2.38
	61.88	72.71 ± 1.34	117.5 ± 3.1	1.87

## Conclusion

In summary, we successfully developed an optosensing probe for spermine, based on fluorescence quenching of the CP-Tb^3+^ complex. Spermine breaks the rigid coordination between CP and Tb^3+^, inducing the quenching of strong green emission of CP-Tb^3+^. The proposed method exhibits a low limit of detection (0.17 μM) with the wide linear range of 2–180 μM. Moreover, the proposed technique was applied on PSTs coupled with a smartphone and Arduino-based device as detection systems with LOD of 1.3 μM and 3.3μM, respectively. The visual detection of the method also showed a potential to detect SP in biological samples.

## Supporting information

S1 File (DOCX)
