# Supplementary material for: Simple fluorescence optosensing probe for spermine based on ciprofloxacin-Tb3+ complexation
Source: PLoS One. 2021 May 10;16(5):e0251306. doi: 10.1371/journal.pone.0251306 (PMC8109780; doi:10.1371/journal.pone.0251306)
Supplement: S1 File — (DOCX) [file pone.0251306.s001.docx]

**S1 File**

**Simple fluorescence optosensing probe for spermine based on ciprofloxacin-Tb^3+^ complexation**

Nguyen Ngoc Nghia^1^, Bui The Huy^1,*^, Pham Thanh Phong^2,3^, Jin Sol Han^1^, Dae Hyun Kwon^1^, Yong-Ill Lee^2,3,*^

^1^Department of Materials Convergence and System Engineering, Changwon National University, Changwon 51140, Republic of Korea

^2^Ceramics and Biomaterials Research Group, Advanced Institute of Materials Science, Ton Duc Thang University, Ho Chi Minh City, Viet Nam

^3^Faculty of Applied Sciences, Ton Duc Thang University, Ho Chi Minh City, Viet Nam

**Characteristic of AS7262 6-Channel VIS Sensor**

**Features**

- Integrated interference filters directly deposited on standard CMOS silicon
- 6 spectral channels spaced at 450, 500, 550, 570, 600 and 650 nm
- Integrated microcontroller on chip and low power consumption
- 2 integrated LED drivers
- Selectable interfaces UART or standard I²C
- in-factory calibrated

**Benefits**

- Stable filter conditions over temperature and life time
- Detection of spectral fingerprints in visible spectrum
- Signal processing on chip, reduced hardware costs
- Sample illumination without external LED driver
- Easy connecting to mobile devices or external µController

| 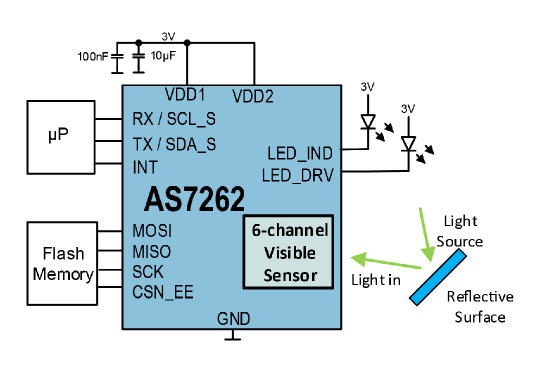 |
| --- |
| **Fig S1**. Detail circuit of AS7262 6 channels VIS sensor. |

Performing experiment

- Connect device with computer by USB cable
- Insert PAD into a slit between AS7262 sensor and UV LED
- Collect data on TFT LCD or using Arduino Software

The detailed electrical connections are as follows:

**2.2'' 320x240 TFT LCD to** **Arduino Uno**

Pin A_0_ (TFT LCD) to pin 9 (Arduino Uno)

Pin CS (TFT LCD) to pin 10 (Arduino Uno)

Pin SDA (TFT LCD) to pin 11 (Arduino Uno)

Pin RESET (TFT LCD) to pin 12 (Arduino Uno)

Pin SCK (TFT LCD) to pin 13 (Arduino Uno)

Pin LED (TFT LCD) to pin 3V3 (Arduino Uno)

Pin VCC (TFT LCD) to pin 5V (Arduino Uno)

Pin GND (TFT LCD) to pin GND (Arduino Uno)

**UV led to Arduino Uno**

Positive charge (UV led) to pin 5V (Arduino Uno) through a resistor (330Ω)

Negative charge (UV led) to pin GND (Arduino Uno)

**AS7262 to Arduino Uno**

Pin Vin (AS7262) to pin 5V (Arduino Uno)

Pin GND (AS7262) to pin GND (Arduino Uno)

Pin SCL (AS7262) to pin A5 (Arduino Uno)

Pin SDA (AS7262) to pin A4 (Arduino Uno)

**Code**

The Arduino is controlled by code written with the open-source Arduino Software (IDE). To use the TFT LCD, AS7262 select Tools/Manage Libraries from menu of the Arduino IDE and then install Adafruit_ST7735, Adafruit_AS726x.h.

| #include <Wire.h>  #include "Adafruit_AS726x.h"  #include <Adafruit_GFX.h> // Core graphics library  #include <Adafruit_ST7735.h> // Hardware-specific library  #include <SPI.h>  // For the breakout, you can use any 2 or 3 pins  // These pins will also work for the 1.8" TFT shield  #define TFT_CS 10  #define TFT_RST 12 // you can also connect this to the Arduino reset  // in which case, set this #define pin to -1!  #define TFT_DC 9  #define SENSOR_MAX 5000  #define BLACK 0x0000  #define GRAY 0x8410  #define WHITE 0xFFFF  #define RED 0xF800  #define ORANGE 0xFA60  #define YELLOW 0xFFE0  #define LIME 0x07FF  #define GREEN 0x07E0  #define CYAN 0x07FF  #define AQUA 0x04FF  #define BLUE 0x001F  #define MAGENTA 0xF81F  #define PINK 0xF8FF  uint16_t colors[] = {  MAGENTA,  BLUE,  GREEN,  YELLOW,  ORANGE,  RED  };  Adafruit_ST7735 tft = Adafruit_ST7735(TFT_CS, TFT_DC, TFT_RST);  //create the object  Adafruit_AS726x ams;  //buffer to hold raw values (these aren't used by default in this example)  //uint16_t sensorValues[AS726x_NUM_CHANNELS];  //buffer to hold calibrated values  float calibratedValues[AS726x_NUM_CHANNELS];  uint16_t barWidth;  uint16_t sensorValues[AS726x_NUM_CHANNELS];  void setup() {    Serial.begin(9600);  tft.initR(INITR_BLACKTAB);  //tft.initR(INITR_MINI160x80); // initialize a ST7735S chip, mini display  tft.setRotation(3);  tft.fillScreen(ST7735_BLACK);    barWidth = tft.width() / AS726x_NUM_CHANNELS;    // initialize digital pin LED_BUILTIN as an output.  pinMode(LED_BUILTIN, OUTPUT);  //begin and make sure we can talk to the sensor  if(!ams.begin()){  Serial.println("could not connect to sensor! Please check your wiring.");  while(1);  }    ams.setConversionType(MODE_2);  //uncomment this if you want to use the driver LED (off by default)  //ams.drvOn();  }    void loop() {    if(ams.dataReady()){    //read the values!  //ams.readRawValues(sensorValues);  ams.readCalibratedValues(calibratedValues);  //read the values!    ams.readRawValues(sensorValues);  //ams.readCalibratedValues(calibratedValues);  Serial.print(" Violet: "); Serial.print(sensorValues[AS726x_VIOLET]);  Serial.print(" Blue: "); Serial.print(sensorValues[AS726x_BLUE]);  Serial.print(" Green: "); Serial.print(sensorValues[AS726x_GREEN]);  Serial.print(" Yellow: "); Serial.print(sensorValues[AS726x_YELLOW]);  Serial.print(" Orange: "); Serial.print(sensorValues[AS726x_ORANGE]);  Serial.print(" Red: "); Serial.print(sensorValues[AS726x_RED]);  Serial.println();  Serial.println();  //tft.fillScreen(ST77XX_BLACK);    tft.setTextColor(ST77XX_MAGENTA);  //tft.setTextSize(1);  //tft.println("VIOLET");  tft.print(sensorValues[AS726x_VIOLET]);    // tft.setCursor(20, 10);  tft.setTextColor(ST77XX_BLUE);  tft.print(sensorValues[AS726x_BLUE]);    tft.setTextColor(ST77XX_GREEN);  tft.print(sensorValues[AS726x_GREEN]);    // tft.setCursor(0, 10);  tft.setTextColor(ST77XX_YELLOW);  tft.print(sensorValues[AS726x_YELLOW]);    tft.setTextColor(ST77XX_ORANGE);  tft.print(sensorValues[AS726x_ORANGE]);    tft.setTextColor(ST77XX_RED);  tft.print(sensorValues[AS726x_RED]);    delay(200);    for(int i=0; i<AS726x_NUM_CHANNELS; i++){  uint16_t height = map(calibratedValues[i], 0, SENSOR_MAX, 0, tft.height());  tft.fillRect(barWidth * i, 0, barWidth, tft.height() - height, ST7735_BLACK);  tft.fillRect(barWidth * i, tft.height() - height, barWidth, height, colors[i]);  }  }  tft.setCursor(60, 0);  tft.setTextColor(ST77XX_WHITE);  //tft.setTextSize(1);  tft.println("ANASTRO");  } |
| --- |

## Information for experimental equipment

Table S1, Table S2 show the tools used and the purpose of their usage through the implementation of the proposed system.

**Table S1**. Hardware tools need for the proposed system.

| **Icon** | **Hardwarde** | **Description** | **Use** |
| --- | --- | --- | --- |
| 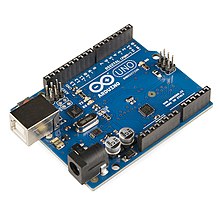 | Arduino Uno R3 | A board based on the ATmega328P microcontroller, having 14 digital input/output pins (6 analog inputs, a quartz crystal of 16 MHz, a USB connection, a power jack, an ICSP header and a reset button) | Programming and connecting the board to control UV LED, photodiode and measure the voltage value from load resistor (R_L_) |
| 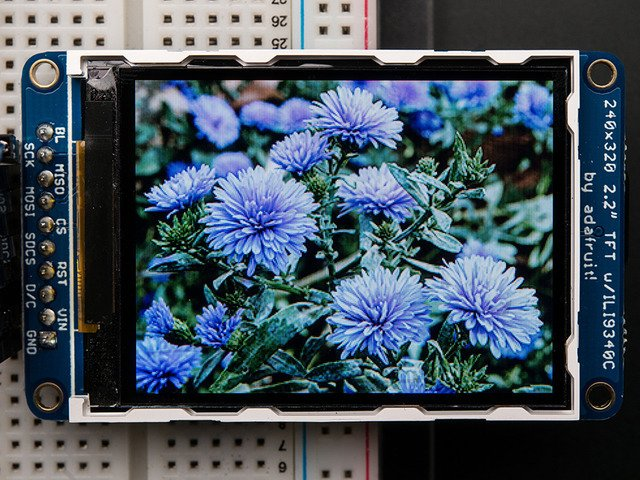 | 2.2” TFT LCD | A thin-film-transistor liquid-crystal display (TFT LCD) is a variant of a liquid-crystal display (LCD) that uses thin-film-transistor (TFT) technology to improve image qualities such as addressability and contrast. A TFT LCD is an active matrix LCD, in contrast to passive matrix LCDs or simple, direct-driven LCDs with a few segments. | Display the values of measurement on it |
| 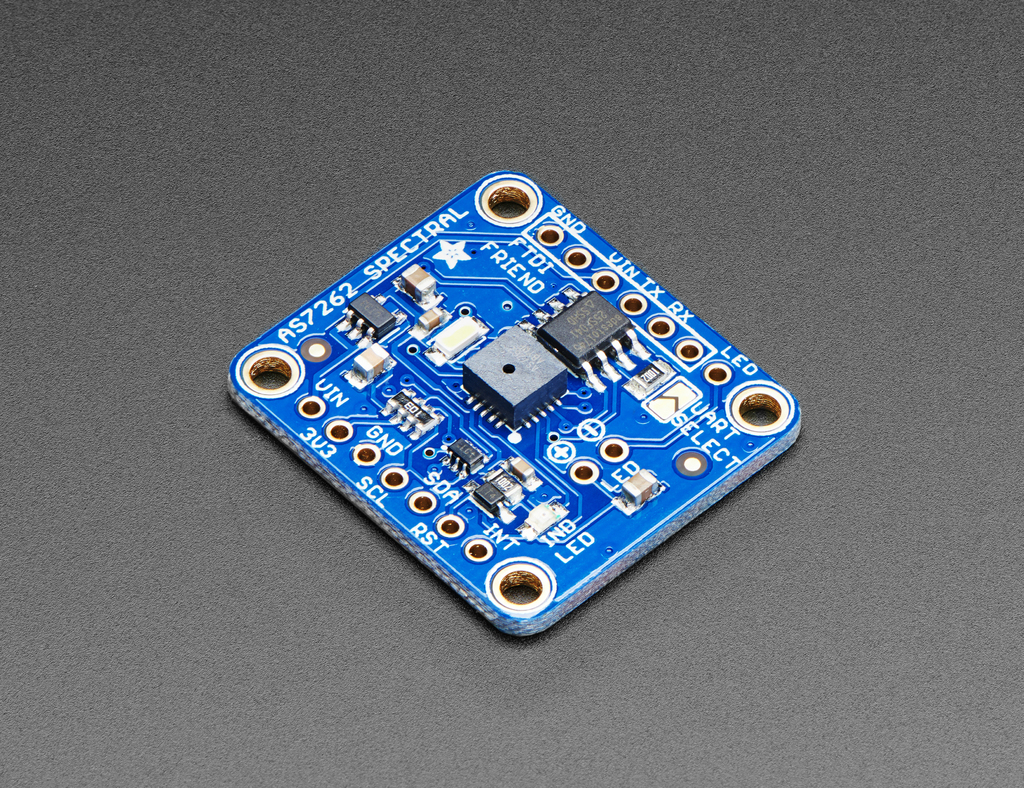 | AS7262 | xxxxx | xxxx |
| 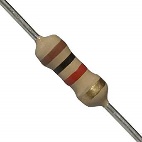 | Resistor 330Ω (R_1_) | A passive two-terminal electrical component | Reduce voltage levels, also current flow in circuits |
| 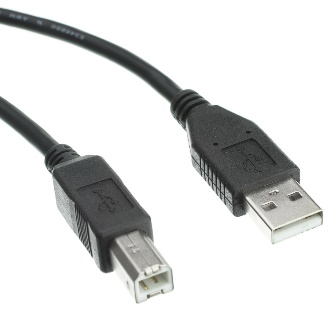 | USB 2.0 cable type A/B | Cable lenght is 178cm | Connect Arduino Uno with the USB female of computer |
| 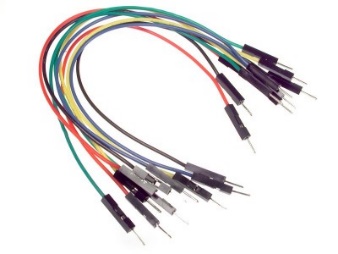 | Cable | An electrical cable consists of two or more wires that run side by side, twisted, or braided together to form a single assembly | Connected to two devices, allowing electrical signals to be transferred between them |

**Table S2. Software tools needed for the proposed system.**

| **Icon** | **Software** | **Description** | **Use** |
| --- | --- | --- | --- |
| 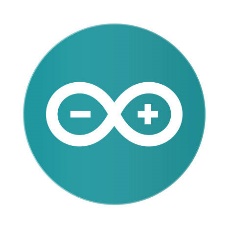 | Arduino Software (IDE) | The open-source Arduino Software (IDE) to write code and upload it to the board, running on Windows, Mac OS X, and Linux. The environment is written in Java | To write the code and upload it on the Arduino board |

| 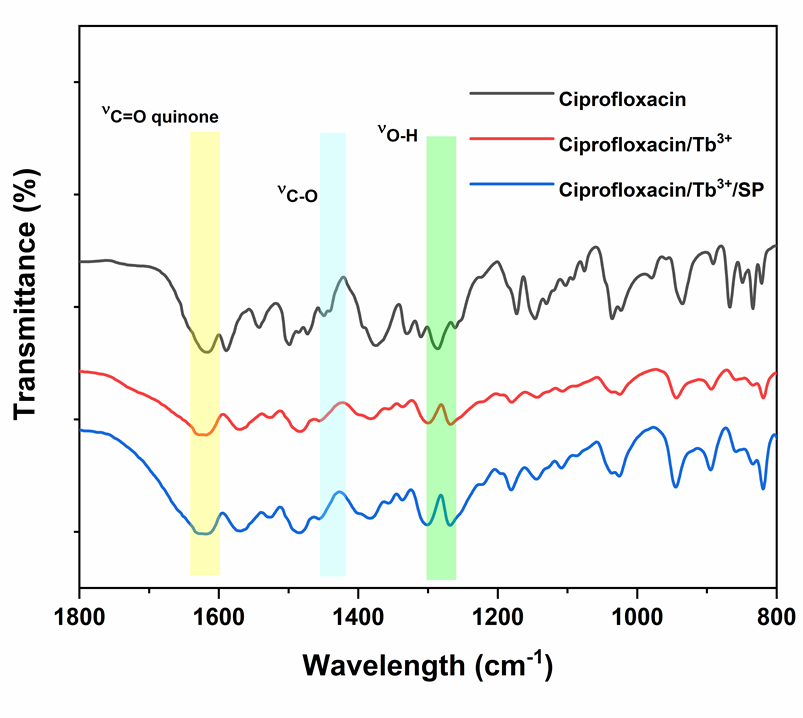 |
| --- |
| **Fig S2**: FTIR spectra of CP, CP-Tb^3+^ and CP-Tb^3+^-SP with CP (0.13 mM), SP (74 µM), Tb^3+^ (0.02 mg/mL). The bending vibration peaks of the O-H group are blue-shifted in the presence of Tb^3+^. |
